# More than ancillary records: clinical implications of renal pathology examination in tumor nephrectomy specimens

**DOI:** 10.1007/s40620-021-01030-0

**Published:** 2021-04-22

**Authors:** Johannes Philipp Kläger, Ahmad Al-Taleb, Mladen Pavlovic, Andrea Haitel, Eva Comperat, Harun Fajkovic, Željko Kikić, Renate Kain, Nicolas Kozakowski

**Affiliations:** 1 Department of Pathology, Medical University of Vienna, General Hospital, Waehringer Guertel 18-20, 1090 Vienna, Austria; 2 Department of Pathology, Mubarak Al-Kabeer Hospital, Jabriya, Kuwait; 3 Department of Urology, Medical University of Vienna, Vienna, Austria; 4 UMRS 1155, Institut National de la Santé et de la Recherche Médicale (INSERM), Tenon Hospital, Paris, France

**Keywords:** Tumor nephrectomy, Renal pathology, Medical kidney disease, EGFR

## Abstract

**Background:**

Nephrectomy is the management of choice for the treatment of renal tumors. Surgical pathologists primarily focus on tumor diagnosis and investigations relating to prognosis or therapy. Pathological changes in non-neoplastic tissue may, however, be relevant for further management and should be thoroughly assessed.

**Methods:**

Here, we examined the non-neoplastic renal parenchyma in 206 tumor nephrectomy specimens for the presence of glomerular, tubulo-interstitial, or vascular lesions, and correlated them with clinical parameters and outcome of renal function.

**Results:**

We analyzed 188 malignant and 18 benign or pseudo-tumorous lesions. The most common tumor type was clear cell renal cell carcinoma (CCRCC, n = 106) followed by papillary or urothelial carcinomas (n = 25). Renal pathology examination revealed the presence of kidney disease in 39 cases (18.9%). Glomerulonephritis was found in 15 cases (7.3%), and the most frequent was IgA nephropathy (n = 6; 2.9%). Vasculitis was found in two cases (0.9%). In 15 cases we found tubulo-interstitial nephritis, and in 9 severe diabetic or hypertensive nephropathy. Partial nephrectomy was not linked to better eGFR at follow-up. Age, vascular nephropathy, glomerular scarring and interstitial fibrosis were the leading independent negative factors influencing eGFR at time of surgery, whereas proteinuria was associated with reduced eGFR at 1 year.

**Conclusion:**

Our large study population indicates a high incidence of renal diseases potentially relevant for the postoperative management of patients with renal neoplasia. Consistent and systematic reporting of non-neoplastic renal pathology in tumor nephrectomy specimens should therefore be mandatory.

**Supplementary Information:**

The online version contains supplementary material available at 10.1007/s40620-021-01030-0.

## Introduction

According to the European Cancer Information System, kidney cancer was the seventh most common and eighth most lethal cancer type in Europe in 2018 [1]. Radical surgical removal is often the treatment of choice, and careful pathological examination of specimens allows precise and predictive diagnosis, classification, and staging. Tumor removal leads to nephron loss, reduced kidney function, and compensatory augmentation of filtration in the remaining nephrons. This promotes progressive loss of kidney function and chronic kidney disease (CKD) [2, 3]. CKD is not only a disabling condition for the affected patients and their families but also represents a high burden for the healthcare systems. Risk factors that contribute to CKD are smoking, obesity, arterial hypertension, and diabetes—the first three have been confirmed and the latter is considered a risk factor for the development of renal cancer [4]. Stratifying patients at risk before surgery aims at reducing the incidence of CKD. However, identification of parenchymal pathologies in the non-tumorous part of the kidney specimens could add crucial data to the risk evaluation of often elderly multi-comorbid patients, and improve postoperative kidney function by an adapted follow-up management [5].

Therefore, we have evaluated the changes in the non-neoplastic renal tissue in more than two hundred specimens of (uretero)-nephrectomy for putative or confirmed kidney or renal pelvic tumors and correlated clinico-pathological findings with kidney function at time of surgery and at one-year follow-up.

## Materials and methods

We prospectively collected non-neoplastic tissue from 129 adult tumor nephrectomy specimens and retrospectively re-evaluated 77 archived cases from our Department. Altogether, 206 cases of radical or partial nephrectomies were collected between March 2013 and March 2019. None of the patients had any intervention not required for clinical care. We performed the study following the Helsinki declaration and with the approval of the local ethics committee (nr. 2020/2019).

We retrieved clinical and laboratory data from the computerized hospital information system or by contacting specialists providing follow-up care. Data included the patient’s demographic information, pre-operative kidney function (estimated glomerular filtration rate (eGFR)) calculated with the CKD-EPI equation, and urine analysis (significant proteinuria defined as more than 150 mg/24 h and sediment). To evaluate follow-up kidney function, we collected eGFR 12 months after surgery, when available. The routine pathological reports provided the diagnosis and classification of tumor lesions and pathological staging. Our detailed nephropathological workup is available in Supplementary Material S1.

We present continuous statistical data as median and interquartile range (IQR), and categorical variables as absolute and relative frequencies. We used Mann Whitney U-tests and Chi-square tests to compare continuous and categorical data, and calculated Pearson’s or Spearman’s coefficients for correlations. Univariate linear regressions allowed the evaluation of renal function in subgroup analyses. After this step, relevant and significant variables (p-value < 0.05) were integrated into multivariate linear regression models, excluding collinear variables. A two-sided p-value of less than 0.05 was considered statistically significant. For statistical analysis, we used Prism 8 for macOS (GraphPad Software, La Jolla, CA, USA), which provided graphs as well, or IBM SPSS Statistics 24 for cross tabs and regression analyses (IBM Corporation, Armonk, NY, USA).

## Results

### Study population

**Fig. 1:**
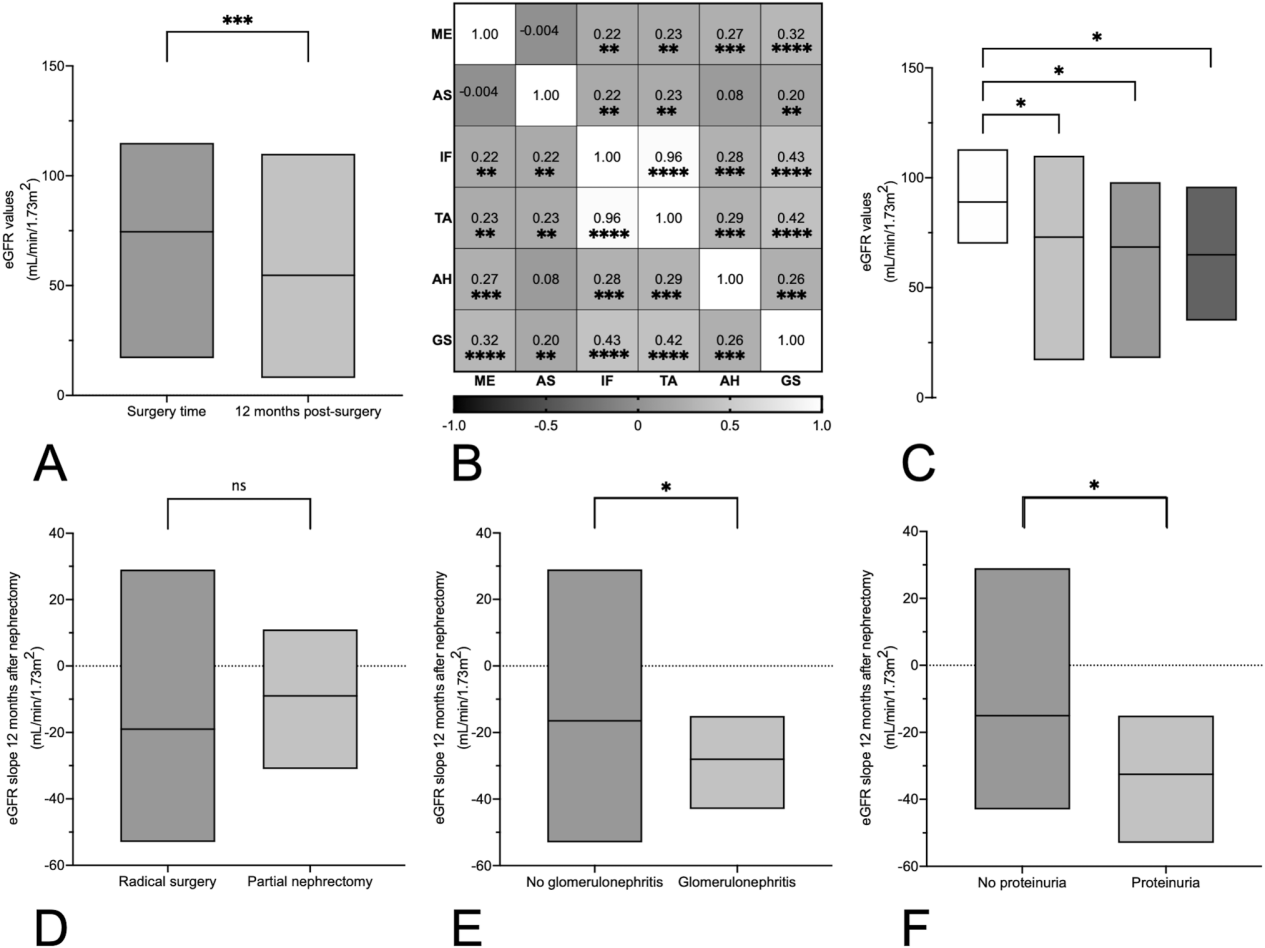
eGFR values or slopes between relevant clinical groups. **a** The eGFR at one-year follow-up is significantly lower than eGFR at the time of surgery. **b** Heat map for Spearman’s correlation r-values shows correlations between histological single lesions. *ME* mesangial expansion, *AS* arteriosclerosis, *IF* interstitial fibrosis, *TA* tubular atrophy, *AH* arteriolar hyalinosis, *GS* glomerular scarring. **c** The eGFR at the time of surgery is lower in the CCRCC, the ICNe and the UrC groups than in the group without neoplasia. **d** The eGFR slope at 12 months does not significantly differ between radial and partial nephrectomy. **e** The eGFR slope at 12 months is significantly lower in case of the coincidental finding of glomerulonephritis at pathology. **f** The eGFR slope at 12 months is significantly lower in case of proteinuria. *ns* not significant; *p < 0.05; **p < 0.01; ***p < 0.001, ****p < 0.0001

Eighty-eight patients (42.7%) were females, with a median age of 70 years (58–76), and 118 were males (57.3%), median age 66 years (59–76). Total or partial nephrectomy was performed in 188 (91.2%) and 18 (8.7%) cases, respectively. Median eGFR at the time of nephrectomy was 73 mL/min/1.73 m^2^ (54–87). Data analysis was corrected for patients with eGFR < 15 mL/min at surgery. We excluded these cases (n = 6) in Table 1 and when using eGFR in statistical evaluation. Proteinuria was present in 26 patients with total and in 2 patients with partial nephrectomy. Ninety-two patients had a follow-up eGFR (90 patients with eGFR > 15 mL/min/1.73 m^2^) which was significantly lower than that at the time of surgery (54 mL/min/1.73 m^2^ (38–68), p < 0.001, Fig. 1a). Patients’ data grouped according to malignancy, tumor subgroups and non-neoplastic conditions are detailed in Tables 1 and 2. Only patients with metastasized renal cell carcinoma (RCC) (33; 16%) received adjuvant therapy, including chemo-, radio-, immuno- or targeted therapy, alone or in combination, while only 3 patients (1.4%) received neoadjuvant therapy for their kidney tumor. Nine (4.4%) further patients received cytoreductive therapy for another malignancy (4 of them before diagnosis and therapy of the renal tumor—details in Table S1). Radiotherapy was exclusively performed in kidney-sparing fields (cerebral, osseous or pelvic).

**Table 1:** Study population, neoplastic, benign and malignant tumors and clinical data

	Study population	Neoplastic Diseases	Malignant tumors	Benign tumors
Nb (nb of benign tumors)	206 (11)	199	188	11
*Clinical data*				
Age in years*	68 (59–76)	69 (60–76)	69 (60–76)	55 (42–67)
			p = .01	
Female (%)	88 (43)	86 (43)	79 (42)	7 (64)
Proteinuria (%)^†^	28 (21; 131)	27 (22; 125)	24 (21; 115)	3 (30; 10)
Arterial hypertension (%)	129 (63)	125 (63)	120 (64)	5 (45)
Diabetes mellitus (%)	59 (29)	57 (29)	56 (30)	1 (9)
eGFR at surgery*	73 (54–87)	73 (54–87)	73 (53–86)	85 (64–98)
eGFR at 12 months*^†^	55 (39–69; 90)	53 (38–66; 87)	53 (38–68; 83)	46 (36–63; 4)
eGFR drop at 12 months*^†^	17 (7–37; 90)	19 (7–31; 87)	18 (7–30; 83)	41 (17–50; 4)

*In median (interquartile range); ^†^from n patients in brackets; *nb* number, *eGFR* estimated glomerular filtration rate in ml/min/1.73 m^2^, CKD-EPI equation. Pearson's Chi squared test and Student's t-test or Mann–Whitney U-test were applied for comparison of categories or means when appropriate

**Table 2:** Tumor subtypes and clinical data

	Non neoplastic diseases	CCRCC	PRCC	ChRCC	UrC	Miscellaneous tumors
Nb (nb of benign tumors)	7	106	29 (4)	20 (4)	25	19 (3)
*Clinical data*						
Age in years*	55 (20–64)	69 (60–76)	61 (54–77)	62 (57–72)	73 (66–80)	50 (37–66)
	p = 0.003					
	p = 0.03					
	p = 0.03					
	p < 0.001					
		p = 0.01				
			p = 0.008			
				p = 0.001		
Female (%)	2 (28)	45 (42)	7 (24)	13 (65)	15 (60)	6 (31)
			p = 0.007			
			p = 0.01			
Proteinuria (%)†	1 (17; 6)	13 (21; 62)	9 (41; 22)	1 (8;12)	2 (11; 18)	2 (18; 11)
Art. hypertension (%)	4 (57)	64 (60)	19 (66)	13 (65)	15 (60)	14 (74)
Diabetes mellitus (%)	2 (28)	39 (37)	4 (14)	3 (15)	8 (32)	3 (16)
		p = 0.024				
eGFR at surgery*	87 (70–97)	73 (58–86)	74 (48–88)	68 (51–90)	64 (49–86)	86 (48–90)
	p = 0.02					
	p = 0.05					
	p = 0.01					
eGFR at 12 months*^†^	77 (54–106; 5)	51 (37–68; 41)	54 (36–57; 17)	52 (39–75; 8)	50 (37–64; 14)	62 (51–71; 8)
eGFR drop at 12 months*^†^	8 (3–16; 5)	22 (7–33; 41)	20 (9–36; 17)	10 (− 9 to 22; 8)	14 (− 14 to 24; 14)	15 (7–27; 8)

*In median (interquartile range); ^†^from n patients in brackets; *nb* number, *CCRCC* clear cell renal cell carcinoma, *PRCC* papillary neoplasm, *ChRCC* neoplasms originating from intercalating cells of distal tubules, *UrC* urothelial carcinoma, *art. hypertension* arterial hypertension; eGFR: estimated glomerular filtration rate in ml/min/1.73 m^2^, CKD-EPI equation. Pearson's Chi squared test and Student's t-test or Mann–Whitney U-test were applied for comparison of categories or means when appropriate

### Pathological report

**Fig. 2:**
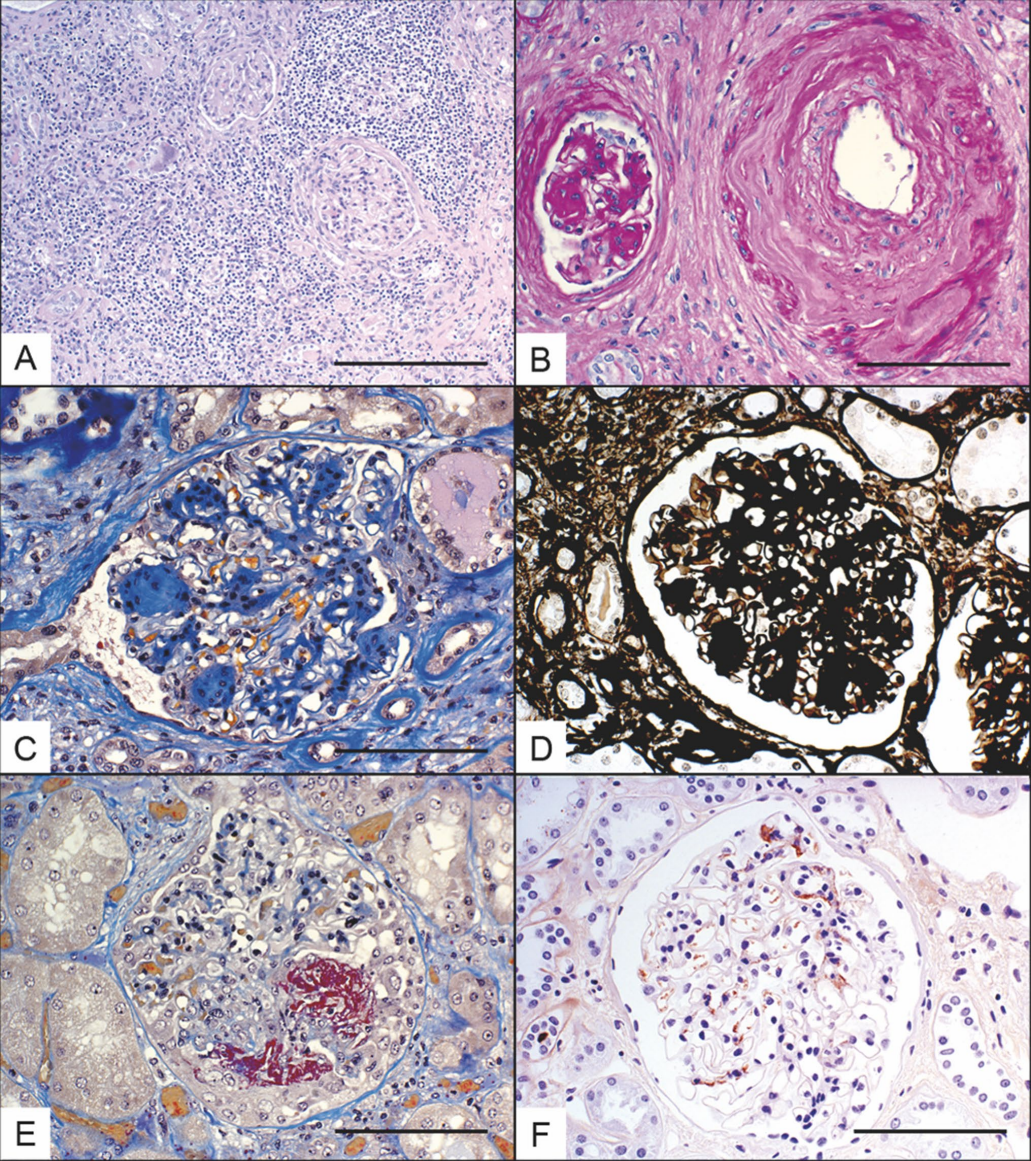
Histopathological lesions found distant from the tumor bed. **a** Interstitial nephritis with a mixed infiltrate of mononuclear cells and plasma cells, in a patient with a urothelial carcinoma G3 of the renal pelvis, pT1b (HE, × 100, scale: 100 µm). **b** High-grade arteriosclerosis in a patient with arterial hypertension, significant mesangial expansion and interstitial fibrosis in the same patient (PAS, × 200, scale: 50 µm). **c** Nodular glomerulosclerosis in a diabetic patient, surrounded by significant interstitial fibrosis and tubular atrophy, in a patient with a clear cell carcinoma G2, pt1b (AFOG, × 200, scale: 50 µm). **d** Nodular glomerulosclerosis, thickened basement membranes, in the same diabetic patient (Methenamine, × 200, scale: 50 µm). **e** Segmental capillary necrosis in red, extracapillary proliferation (cellular crescent), with somewhat retracted adjacent capillary loops, in a patient with a positive ANCA serology and a composite tumor of oncocytoma and chromophobe carcinoma G3, pT1b (AFOG, × 200, scale: 50 µm). **f** Positive immunohistochemical stain for IgA with a brown mesangial signal, in a patient with a clear cell carcinoma G2, pT3a (DAB & counterstain in HE, × 200, scale: 50 µm)

Tumor details and nephropathological findings are reported in Table 3. We found additional kidney diseases in 39 patients (18.9%). Six patients (2.9%) in our cohort had IgA-nephropathy, 2 (0.9%) glomerulonephritis related to ANCA-associated vasculitis (AAV), 7 (3.4%) immune complex-mediated (other than IgA) glomerulonephritides, five advanced diabetic nephropathies (2.4%), 15 tubulointerstitial nephritides ([TIN]; 7.3%) of different nature (i.e., xanthogranulomatous or chronic interstitial nephritides, 4 of them locally developing pseudo-tumorous lesions, or of undetermined etiology), and 4 (1.9%) hypertensive nephropathy. Three further patients displayed recanalized arterial thrombi or onion-skinning of arteriolar media evocative of chronic thrombotic microangiopathy;  together with the cases of hypertensive nephropathy these lesions accounted for 3.4% of the cases in our cohort. Only one case had both diabetic changes and TIN, the latter feature being prominent and considered the leading and classifying non-neoplastic lesion. Lesions in individual compartments collectively indicating chronic changes, like scarring (mesangial expansion, tubular atrophy, interstitial fibrosis, arteriosclerosis, and arteriolar sclerosis/hyalinosis—examples in Fig. 2) were semi-quantitatively examined and correlated well with each other (Fig. 1b). Of note, interstitial fibrosis and tubular atrophy showed strong correlation (r = 0.98, p < 0.0001). Hence, they have been looked a single pathology in the following analyses (interstitial fibrosis implying “with tubular atrophy”). While the pathology findings are split into malignant and benign disease in Table 4 or into tumor subtypes in Tables 5, 6 describes nephropathological diagnoses in tumor subtypes.

**Table 3:** Details on tumor diagnoses and incidental kidney diseases

	*N* (%)		*N* (%)
Kidney neoplasms	199 (96.6%)	Kidney diseases	39 (18.9)
Clear cell renal cell carcinoma	106 (51.4)	Glomerular diseases	20 (9.7)
		*Diabetic nodular glomerulosclerosis*	5 (2.4)
Papillary neoplasms	29 (14.1)	*IgA-nephritis*	6 (2.9)
*Papillary carcinoma*	25 (10)	*ANCA-associated vasculitis*	2 (0.9)
*Papillary adenoma*	4 (2)	*Immune complex glomerulonephritis*	7 (3.4)
Neoplasms originating from intercalating cells of distal tubuli	20 (10)	Vascular diseases	
*Chromophobe carcinoma*	16 (8)	*Hypertensive nephropathy*	4 (1.9)
*Oncocytoma*	4 (2)		
		Tubulo-interstitial diseases	15 (7.3)
Urothelial carcinoma	25 (12.1)	*Xantho-granulomatous nephritis*	1 (0.4)
		*Chronic pyelonephritis*	5 (2.4)
Miscellaneous renal neoplasms	19 (6.3)	*Sarcoidosis*	1 (0.4)
*Collecting duct carcinoma*	2 (1)	*Acute pyelonephritis*	1 (0.4)
*Cystic disease associated RCC*	6 (3)	*Renal abscess*	1 (0.4)
*Mesonephric adenosarcoma*	1 (0.5)	*Interstitial nephritis*	6 (2.9)
*Hemangioblastoma*	1 (0.5)		
*Leiomyosarcoma*	1 (0.5)		
*Metastasis*	1 (0.5)		
*Multilocular cystic RCC*	2 (1)		
*Mucinous tubular and spindle cell carcinoma*	1 (0.5)		
*Translocation carcinoma*	1 (0.5)		
*Angiomyolipoma*	1 (0.5)		
*Nephroblastoma*	1 (0.5)		
*Pecoma*	1 (0.5)		

*ANCA* anti-neutrophilic cytoplasmic autoantibody, *RCC* renal cell carcinoma

**Table 4:** Study population, neoplastic, benign and malignant tumors and pathological findings

	Study population	Neoplastic diseases	Malignant tumors	Benign tumors
Nb (nb of benign tumors)	206 (11)	199	188	11
*Pathology data*				
Partial nephrectomy (%)	18 (9)	17 (9)	17 (9)	0 (0)
Stage in malignant cases^*†^	n.a	n.a	3 (1–3; 167)	n.a
Glomerulonephritis (%)	15 (7)	15 (8)	14 (7)	0 (0)
Vasc. nephropathy (%)	9 (4)	9 (5)	8 (4)	1 (9)
Tub.-int. nephritis (%)	15 (7)	11 (6)	10 (5)	1 (9)
*Histology lesions*				
Mesangial sclerosis*	0 (0–1)	0 (0–1)	0 (0–1)	0 (0–1)
Arteriosclerosis*	2 (1–3)	2 (1–3)	2 (1–3)	2 (1–2)
Arteriolosclerosis^*^	0 (0–0)	0 (0–0)	0 (0–0)	0 (0–1)
Interstitial fibrosis*	0 (0–1)	0 (0–1)	0 (0–1)	1 (0–2)
Glomerular scarring (%)*	2 (0–5)	2 (0–5)	2 (0–5)	4 (0–10)

*In median (interquartile range); ^†^from n patients in brackets; *nb* number, *n.a.* not applicable, *tub.-int. nephritis* tubulointerstitial nephritis, *vasc. nephropathy* vascular nephropathy. Pearson's Chi squared test and Student's t-test or Mann–Whitney U-test were applied for comparison of categories or means when appropriate. For histology lesions, each parameter but glomerular scarring was semi-quantitatively scored 0–3 (none, mild, moderate, severe)

**Table 5:** Tumor subtypes and pathological findings

	Non-neoplastic diseases	CCRCC	PNe	ICNe	UrC	Miscellaneous tumors
Nb (nb of benign tumors)	7	106	29 (4)	20 (4)	25	19 (3)
*Pathology data*						
Partial nephrectomy (%)	1 (14)	10 (9)	3 (10)	3 (15)	0 (0)	1 (5)
Stage in malignant cases^*†^	n.a	3 (3–3; 96)	1 (1–3; 23)	1 (1–3; 14)	2 (1–3; 22)	3 (1–3; 13)
		p < 0.001				
		p < 0.001				
		p = 0.003				
Glomerulonephritis (%)	0 (0)	5 (5)	5 (17)	1 (5)	0 (0)	4 (21)
		p = 0.02				
Vasc. nephropathy (%)	0 (0)	4 (4)	2 (7)	0 (0)	1 (4)	2 (11)
Tub.-int. nephritis (%)	4 (57)	6 (6)	1 (3)	0 (0)	2 (8)	2 (11)
Histology lesions						
Mesangial sclerosis*	0 (0–0)	0 (0–1)	0 (0–1)	0 (0–1)	0 (0–1)	1 (0–1)
Arteriosclerosis*	2 (1–3)	2 (1–3)	2 (1–3)	2 (1–3)	2 (1–3)	2 (1–3)
Arteriolosclerosis*	0 (0–0)	0 (0–0)	0 (0–0)	0 (0–0)	0 (0–0)	0 (0–1)
Interstitial fibrosis*	1 (0–3)	0 (0–1)	0 (0–1)	0 (0–1)	1 (0–2)	1 (0–1)
	p = 0.04					
	p = 0.03					
		p = 0.05				
			p = 0.03			
Glomerular scarring (%)*	1 (3–15)	1.5 (0–5)	2 (0–6)	1 (0–2)	2 (1–5)	3 (0–15)

*In median (interquartile range); ^†^from n patients in brackets; *nb* number, *n.a.* not applicable, *CCRCC* clear cell renal cell carcinoma, *PNe* papillary neoplasm, *ICNe* neoplasms originating from intercalating cells of distal tubules, *UrC* urothelial carcinoma, *tub.-int. nephritis* tubulointerstitial nephritis, *vasc. nephropathy* vascular nephropathy. Pearson's Chi squared test and Student's t-test or Mann–Whitney U-test were applied for comparison of categories or means when appropriate. For histology lesions, each parameter but glomerular scarring was semi-quantitatively scored 0–3 (none, mild, moderate, severe)

**Table 6:** Tumor subtypes and nephropathological diagnoses

	Study population	Non-neoplastic diseases	CCRCC	PNe	ICNe	UrC	Miscellaneous tumors
Nb (nb of benign tumors)	206 (11)	7	106	29 (4)	20 (4)	25	19 (3)
No supplementary relevant nephropathological finding	167 (81.1)	3 (42.9)	91 (85.8)	21 (72.4)	19 (95)	22 (88)	11 (57.9)
IgA nephritis	6 (2.9)	0	3 (2.8)	2 (6.9)	0	0	1 (5.3)
Frank diabetic nephropathy	5 (2.4)	0	4 (3.8)	0	0	1 (4)	0
Interstitial nephritis	15 (7.3)	4 (57.1)	6 (5.7)	1 (3.4)	0	2 (8)	2 (10.5)
Vasculitis	2 (1)	0	0	0	1 (5)	0	1 (5.3)
Immune complex glomerulonephritis	7 (3.4)	0	2 (1.9)	3 (10.3)	0	0	2 (10.5)
Hypertensive nephropathy	4 (1.9)	0	0	2 (6.9)	0	0	2 (10.5)

In number and frequency (%). *nb* number, *CCRCC* clear cell renal cell carcinoma, *PNe* papillary neoplasm, *ICNe* neoplasms originating from intercalating cells of distal tubules, *UrC* urothelial carcinoma

Electron microscopy was performed in 23 cases (11.2%). While mesangial matrix augmentation and thickened basement membranes of the glomerular capillaries supported the diagnosis of diabetic nephropathy, electron-dense deposits at higher magnification confirmed the immune complex-based glomerulonephritides.

### Correlations between clinical data, tumor type, and pathology

Patients were significantly older in the group with neoplastic diseases than in the group with non-neoplastic diseases (69 years (60–76) vs. 55 (20–64), p = 0.002), irrespective of individual tumor subgroup (all p-values < 0.05), and were older in the group with malignant than in the group with benign tumors (69 (60–76) vs. 55.2 (47–71), p = 0.01—Table 1). Age-dependent, intermediate- to high-grade arteriolosclerosis was observed throughout our cohort (Table 2).

All cases of glomerulonephritides occurred in patients with malignant tumors (n = 15, 8%). Glomerulonephritis was associated with less arteriosclerosis (1 (1–2) vs. 1 (0–2), p = 0.04), but was not associated with age, degree of mesangial expansion, glomerular sclerosis or any of the other histologic lesions we studied of our histologic lesions.

There was no statistically significant difference in any single histological lesion when comparing malignant to benign tumors, but more extensive interstitial fibrosis and tubular atrophy was observed in non-neoplastic diseases than in clear cell renal cell carcinoma (CCRCC) or chromophobe renal cell carcinoma (ChRCC) (1 (0–3) vs. 0 (0–1) vs. 0 (0–1), p = 0.04 and p = 0.03, respectively). This was primarily attributable to the four pseudo-tumorous interstitial nephritides among the seven tumor-like conditions. Vascular nephropathy and TIN were not associated with any tumor subgroup or differed when comparing benign and malignant tumors, although TIN was highly represented in the non-neoplastic conditions.

### Prospective correlation to renal parameters at the time of surgery

Age was strongly correlated with eGFR at the time of nephrectomy (r = − 0.45; p < 0.001) and kidney function at nephrectomy was better in the non-neoplastic group than in the groups of CCRCC, papillary neoplasms (PNe), neoplasms originating from intercalating cells of distal tubules (ICNe), or urothelial carcinoma (UrC) (eGFR in mL/min/1.73 m^2^: 87 (70–97) vs. 73 (58–86) vs. 74 (48–88) or 68 (51–90) or 64 (49–86), all p ≤ 0.05, Table 1 and Fig. 1c). Patients with PNe were more often proteinuric (41%), but this did not reach statistical significance. Glomerulonephritis was not associated with a worse eGFR at the time of surgery.

**Fig. 3:**
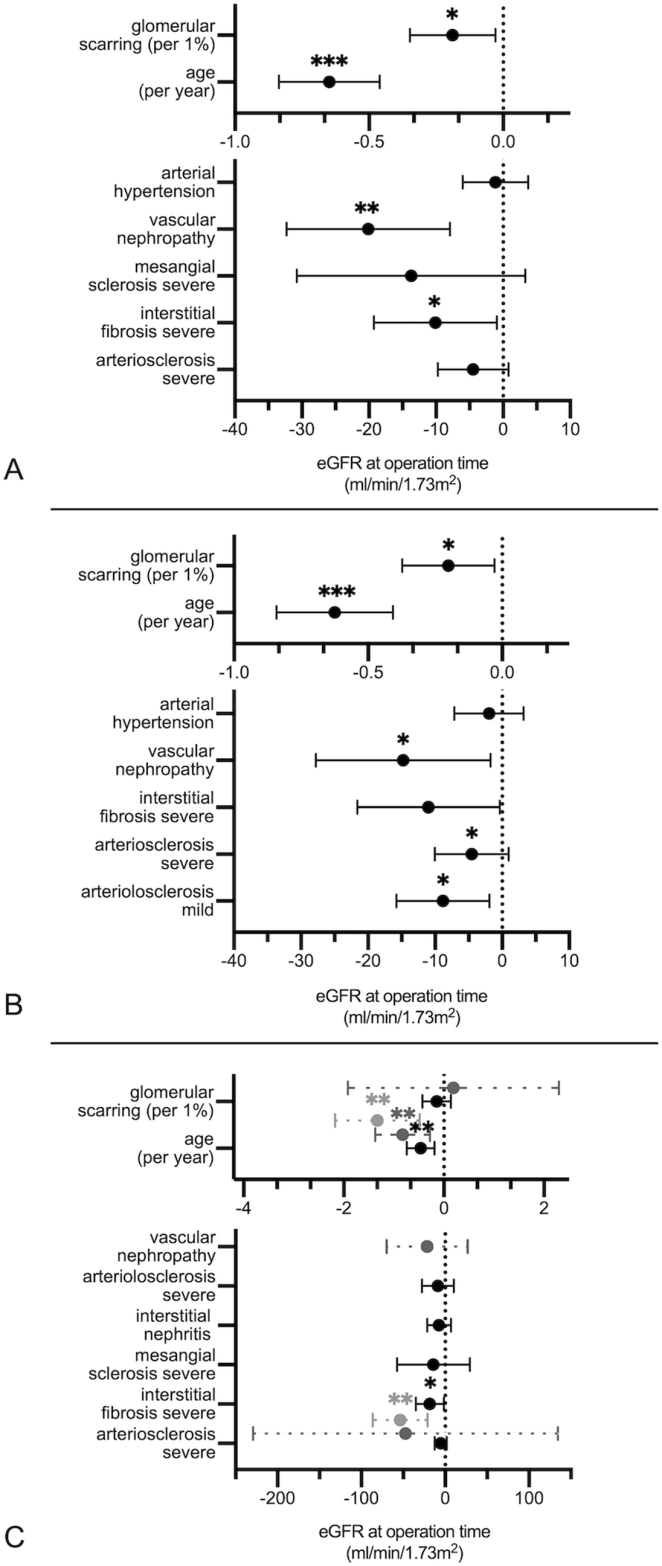
Forest plots of relevant regression models. We computed simple and multiple linear regressions predicting renal function stepwise. Simple regression first included clinical data (age, sex, proteinuria, arterial hypertension, diabetes mellitus), then nephropathological findings (glomerulonephritis, interstitial nephritis and vascular nephropathy), and lastly histological single lesions (glomerular scarring, mesangial expansion, arteriosclerosis, arteriolosclerosis, interstitial fibrosis but without tubular atrophy, which is strongly collinear to it). **a** Multivariate linear regression analysis for eGFR at the time of surgery for the whole cohort (constant = 121 ml/min/1.73m^2^ of eGFR). **b** Multivariate linear regression analysis for eGFR at the time of surgery for malignant tumors (constant = 120.5 ml/min/1.73m^2^ of eGFR). **c** Multivariate linear regression analyses for eGFR at the time of surgery for the CCRCC group (in black; constant = 110.6 ml/min/1.73m^2^ of eGFR), the PNe group (in long dashed dark grey; constant = 137.6 ml/min/1.73m^2^ of eGFR), and the ICNe group (in long dashed light grey; constant = 157.9 ml/min/1.73m^2^ of eGFR). *CI* confidence interval, *ns* not significant; *p < 0.05; **p < 0.01; ***p < 0.001

For the whole cohort, the strongest correlations with eGFR at surgery were with age, vascular nephropathy, glomerular scarring and severe interstitial fibrosis (respectively, β = − 0.649 per year of age, β = − 20.1, β = − 0.189 per % of glomerular sclerosis and β = − 10.1—Fig. 3a and Table S2) in multivariate analysis (F(7,190) = 16.171, p < 0.001, R^2^ = 0.35). Neither adding malignancy of the tumor (F(8,189) = 14.238, p < 0.001, R^2^ = 0.35), nor restricting the analysis to the group of malignant diseases modified the model considerably (F(7,173) = 14.248, p < 0.001, R^2^ = 0.366; Fig. 3b and Table S3). For the latter group, age, vascular nephropathy and severe interstitial fibrosis were the strongest negative factors influencing eGFR at the time of surgery (respectively, β = − 0.624 per year of age, β = − 14.8 and β = − 11).

For each tumor group, the multivariate analysis (provided in Supplementary Tables S4–7) identified various negative factors: age and severe interstitial fibrosis for CCRCC (respectively, β = − 0.463 per year and β = − 18.4, F(8,96) = 7.261, p < 0.001, R^2^ = 0.377, Fig. 3c and Table S4), age for PNe (β = − 0.83 per year, F(4,22) = 5.175, p = 0.004, R^2^ = 0.696, Fig. 3c and Table S5), age and severe interstitial fibrosis for ICNe (β = − 1.3 per year of age and β = − 53.2, F(2,15) = 11.9, p = 0.001, R^2^ = 0.613, model (c)—Fig. 3c and Table S6). Univariate linear regression analysis identified vascular nephropathy as a negative factor for baseline eGFR (β = − 48.9, p = 0.026, R^2^ = 439) in benign lesions). None of the other subgroup analyses provided further meaningful insights.

### Postoperative nephrological assessment

Regular follow-up was performed in the majority of cases on an outpatient basis. Details were therefore available for only about one-third of patients (36%) with other relevant nephropathological diagnoses. Data on nephrological care for the other patients were not available, partly because postoperative care was not performed in our institution. One patient with IgA nephritis received an angiotensin-converting enzyme (ACE) inhibitor, one patient with interstitial nephritis was treated with corticosteroids, and one patient with vasculitis received corticosteroids and rituximab. Seven patients required dialysis after nephrectomy, one because of surgery on a single kidney, two because of double nephrectomy and one because of transplant-nephrectomy. Two patients were already on dialysis at the time of surgery. Four further patients received a kidney allograft during follow-up. Five patients died within the first postoperative year. All of these were excluded from the follow-up analysis of eGFR at one year.

### Prospective correlation to renal parameters at 12 months post-surgery

Age correlated well with eGFR at 12 months follow-up (R = − 0.35, p < 0.001, Figure S1). Partial nephrectomy was associated with a non significantly better follow-up eGFR (69 ml/min/1.73m^2^ (43–89) vs. 55 (40–68) for nephrectomy; p = 0.16) and a trend towards a lesser eGFR decline (9 ml/min/1.73m^2^ (1–21) vs. 20 (8–33); p = 0.11; Fig. 1d). There was no difference between tumor groups for eGFR values or slope at 12 months. The group with glomerulonephritides had a significantly higher eGFR decline at 12 months (28 (19–43) vs. 17 (5–31), p = 0.04; Fig. 1e).

Proteinuria was the sole significant factor negatively influencing the eGFR slope at 12 months post-surgery in linear regression analysis (β = − 19.38, p = 0.003, R^2^ = 0.15—Table S8). Accordingly, the group with proteinuria had a significantly higher eGFR decline at 12 months (32 (19–48) vs. 15 (3–27), p = 0.004; Fig. 1f). Because of potential nephrotoxicity of some neo- or adjuvant therapies (as detailed in Table S1), we performed the same analysis excluding patients who had received such therapies. In this model, proteinuria continued to be significantly correlated to a higher eGFR decline at one year (β = − 19.1, p = 0.003, R^2^ = 0.123), though this stayed true for malignant diseases alone (β = − 14.4, p = 0.047, R^2^ = 0.075—Table S9). No significant factor was identified in further tumor subgroup analysis (data not shown).

## Discussion

Studies on the pathology of non-neoplastic kidney diseases in tumor nephrectomy specimens are scarce in European populations, and the size of the cohort studied  is comparable to the largest ones from other continents [6–8]. Our study provides new data based on a European population with its particular genetic, environmental, socio-economic backgrounds and varying incidence of conditions associated with RCC, such as diabetes or hypertension. Many teams currently strive to improve the identification of patients at risk of reduced kidney function after renal surgery and establishing prediction models mostly based on clinical data [9, 10]. The inclusion of pathology findings could, in our view, ameliorate the predictive power of such tools leading to the discovery of additional relevant kidney lesions  in a noticeable number of patients. Additional glomerular, tubulo-interstitial or vascular nonneoplastic diseases were observed distant from the putative tumor bed in 18.9% of our cases. This incidence is in line with most published data (15–29.4%), which depend, though, on their specific pathologic definition [6–8, 11]. This frequency may seem high in comparison to the general population, but a substantial part of the lesions we described here are related to diabetes mellitus, hypertension, or TIN cases, disorders that are recognized risk factors for RCC. A further common risk factor is the relatively high age of this RCC population, which was an independent adverse influencing factor on eGFR at the time of surgery and was associated with a more substantial eGFR decline at one year, like in other reports.

Taking a closer look at the glomerular compartment, we reported 15 cases of glomerulonephritis, of which one third benefited from nephrological supervision and specific treatments. Diagnosis of glomerulonephritis was associated with an accelerated eGFR decline at 12 months follow-up and was only associated with malignant renal tumors. Here, paraneoplastic glomerular disease could be postulated and this is well described for vasculitis, IgA nephropathy, membranous nephropathy, or membranoproliferative glomerulonephritis in different cancers [12–14]. Nevertheless, we cannot totally rule out a coincidental association. The second glomerular lesion was nodular or diffuse glomerulosclerosis attributed to diabetes in 4.3% of our cases, which is in accordance with the worldwide prevalence of diabetes of 8.5%, where only 20–40% of patients will develop renal complications [15, 16]. All these glomerular lesions are frequent causes of proteinuria, which we identified to be a significant negative factor for follow-up eGFR at one year. In another study, proteinuria was associated with chronic renal lesions at the time of surgery, supporting the deleterious influence we identified in our study [17]. In our work, we were able to take the influence of any (neo-)adjuvant and potentially nephrotoxic therapy into account for the evaluation of postoperative renal function. This had been a difficulty other colleagues encountered in earlier studies [6–8, 18, 19]. Thus, in the context of a putative kidney tumor, the presence of proteinuria should prompt a thorough nephrological investigation for glomerular disease.

We discovered another lesion in 7.3% of our cases: TIN of different nature and varying degrees of associated parenchymal destruction. The pathophysiology of TIN is only partly understood and remains, in many cases, of unknown origin [20]. Both glomerulonephritis and TIN can lead to progressive glomerular scarring, interstitial fibrosis and tubular atrophy. Unsurprisingly, they were independent and significant negative factors for eGFR at surgery, as reported in other studies [8, 17, 21–23]. The same held true for vascular nephropathy, which was also associated with reduced eGFR at surgery. Hypertension and diabetes are the principal causes for such changes, and predictors of eGFR decrease [4, 18, 23–26]. Consequently, describing these lesions in the pathology report, regardless of their cause, should be mandatory as they could be a hurdle for the use of a potentially nephrotoxic adjuvant chemo- or targeted therapy.

Comparing our results with the literature, we can confirm the findings of Bazzi et al., who showed that in 800 partial nephrectomy cases, vascular sclerosis was associated with preoperative eGFR [21], or Malkoc et al. who described relevant findings in 63.5% of 394 specimens of partial nephrectomy, mostly related to co-morbidities and to baseline eGFR [19]. Studies correlating pathological findings of the non-tumoral tissue with follow-up renal function after radical nephrectomy are scarce. The larger international cohorts did not assess this topic at all or not thoroughly ( employing only serum creatinine values) [7, 8, 27]. Our study could, for the first time, evaluate eGFR at 12 months post-surgery and identify proteinuria as a negative predictive factor for kidney function.

There are some limitations to our study. Its partially retrospective character rendered the collection of data some time after surgery challenging. In fact, many of our patients had follow-up medical care in differnt settings, resulting in incomplete eGFR data at 12 months follow-up. However, the prospective and retrospective cohorts had a similar proportion of data available, arguing against a systematic bias. Furthermore, subgroup analyses are sometimes limited. These restrictions may weaken our exploration for predictive pathological lesions. However, our study cohort is, to our knowledge, the largest European and the second largest worldwide study with radical nephrectomies and follow-up data at one year. In this perspective, and in comparison to other studies using serum creatinine as a proxy for renal function, our study provides more granular data, as we took into account eGFR and evaluated it by multivariate analyses [6, 8, 21].

All these predictive findings can hardly be detected if thorough nephropathological evaluation is neglected as it sometimes reveals occult renal diseases of critical importance for the future renal function. The evaluation of non-neoplastic kidney parenchyma should, therefore, be done in a very systematic way and with a high degree of attention. Of note, HE-stained sections are, in our opinion, insufficient to detect all the changes mentioned in this article, thus, we recommend adding at minimum a PAS and a silver stain as an initial evaluation step. Another feasible approach for the surgical pathologist would be to seek out a nephropathologist’s opinion. Especially in centers with limited exposure to kidney diseases, detecting subtle glomerular changes may require a trained “eye” and lead to further ancillary studies such as immunohistochemistry or electron microscopy.

In conclusion, pathological evaluation of the non-neoplastic renal tissue distant from the tumor focus in nephrectomy specimens provides essential data for the surveillance of these patients frequently affected by multiple comorbidity. Pathological evaluation may  reveal occult renal diseases of critical importance for the future renal function, and, subsequently, for the oncologic follow-up and overall prognosis.

## Supplementary Information

Below is the link to the electronic supplementary material.

Supplementary file1 (DOCX 85 KB)

Supplementary file2 (TIFF 23923 KB)
