# Supplementary material for: More than ancillary records: clinical implications of renal pathology examination in tumor nephrectomy specimens
Source: J Nephrol. 2021 Apr 22;34(6):1833–44. doi: 10.1007/s40620-021-01030-0 (PMC8610937; doi:10.1007/s40620-021-01030-0)
Supplement: Supplementary file 1 — Supplementary file1 (DOCX 85 KB) [file 40620_2021_1030_MOESM1_ESM.docx]

S1 – Nephropathological work-out of the non-neoplastic renal parenchyma

Tissue probes consisted of formalin-fixed and paraffin-embedded (FFPE) tissue, taken during routine specimen macroscopic inspection the furthest possible or at least one centimeter away from the tumor, in order to avoid misinterpretation of tumor-induced changes. Staining included hematoxylin and eosin (HE), a periodic acid-Schiff (PAS), a silver stain (methenamine), and the trichrome stain Acid Fuchsine-Orange G (AFOG) from 4μm thick sections. For each case, routine immunohistochemistry consisted in an antibody panel to immunoglobulins and complement (IgG, IgM, IgA, C3c, and C1q) and, if needed, to kappa and lambda light chains. In a subset of cases electron microscopy, either using de-waxed paraffin-embedded or buffered formalin-fixed tissue was performed. The decision on ordering this examination was indication-based, as decided by the reporting nephropathologists, mostly to decipher or confirm immune deposits or basal membrane modifications.

Systematical examination reported the kidney compartments and changes in glomeruli, tubules, interstitium, and blood vessels. Mesangial expansion, tubular atrophy, interstitial fibrosis, arteriosclerosis, and arteriolar sclerosis/hyalinosis were scored semi-quantitatively as none (=0), mild (=1) to moderate (=2) or severe (=3); glomerular scarring in percent. Glomerulonephritis, defined by either mesangial or endocapillary hypercellularity, capillary loop necrosis and/or crescent formation, or immune deposits revealed in immunohistochemistry, was categorized according to international classifications. A minimum of moderate inflammatory tubulointerstitial inflammation, together with a proper clinical context, allowed us to define tubulointerstitial nephritis (TIN) with a compelling level of certainty. We provided a sub-classification of TIN according to the specific lesions found, when possible. Diabetic nephropathy corresponded to diffuse or nodular glomerulosclerosis, together with a clinical history of diabetes. High-grade stenosing arterio-arteriosclerosis, signs of acute or chronic thrombotic microangiopathy (arteriolar and/or glomerular thrombi, onion skin changes of arteriolar tunica media, old and organized and/or recanalized lesions), glomerulosclerosis and interstitial sclerosis fitting to the grade of arterial hypertension allowed us to diagnose hypertensive nephropathy. For further analysis, we aggregated substantial diabetic and hypertensive nephropathy to a more general “vascular nephropathy” group.

Table S1 – Neo- and adjuvant therapy of the cohort, and their potential renal toxicity

| \| **chemotherapy** \| \| **toxic**  **potential** \| **+/-**  **neoadjuvant therapy** \| \| \| --- \| --- \| --- \| --- \| --- \| \| n=  7 \| cisplatin-based (+ gemcitabin (n=5 (+ bone radiotherapy (n=1)), or docetaxel (n=1) or etoposide (n=1^a^)) \| AKI, hypomagne-saemia, renal acidosis[1,2] \| n=  3 \| cisplatin-based (alone^c^ or + gemcitabin (n=1), or docetaxel (n=1)) \| \| 2 \| carboplatin-based (+ gemcitabin (n=1); + etoposide (n=1^b^)) \| AKI, TMA[1,2] \|  \|  \| \| 1 \| capecitabine (+ deno-sumab + sunitinib at 6Mo) \| CI by reduced renal function[3] \|  \|  \| \| 1^d^ \| docetaxel \| AKI[4] \|  \|  \| \| 4 \| mitomycin instillation \|  \|  \|  \| | \| **(multi-) targeted receptor tyrosine kinase inhibitor** \| \| **toxic**  **potential** \| \| --- \| --- \| --- \| \| n=  6 \| sunitinib (+ bone radiotherapy (n=1), or denosumab (n=1)) \| P, H[3,1,2], TI nephritis[5] \| \| 1 \| sunitinib + cabozantinib + bone radiotherapy \| P, H, TI nephritis[3] \| \| 2 \| sunitinib + pazopanib (+ bone radiotherapy (n=1) \| P, H[6] \| \| 1 \| sunitinib + pazopanib + axitinib \| P, H[1,2] \| \| 1 \| cabozantinib \| P, H[3,1] \| \| 1 \| pazopanib \| P, H[6,1] \| \| 1 \| axitinib \| P, H[1,2] \| \| 1 \| tivozanib \| P, H[7] \| | \| **immunotherapy + mRTKi or RxT** \| \| **toxic**  **potential** \| **+/- neoadjuvant therapy** \| \| \| --- \| --- \| --- \| --- \| --- \| \| n=  1 \| sunitinib + nivolumab \| AKI, P, H, TI nephritis [1,8] \|  \|  \| \| 1 \| sunitinib + nivolumab + tivozanib \|  \|  \|  \| \| 1 \| sunitinib + nivolumab + cabozantinib + cerebral radiotherapy \|  \|  \|  \| \| 1 \| nivolumab \|  \|  \|  \| \| 1 \| nivolumab + ipilimumab + cabozantinib \| AKI, P, TI nephritis[1,2] \|  \|  \| \| 1 \| pembroli-zumab + lenvatinib \| AKI, P, H[1,2] \|  \|  \| \| 1 \| pembroli-zumab + cerebral radiotherapy \| AKI, TI nephritis[8] \| 1 \| cisplatin + gemcita-bin \| | \| **other therapy** \| \| **toxic**  **potential** \| \| --- \| --- \| --- \| \| n=  1^d^ \| degarelix + leuprorelin acetate + pelvic radiotherapy \|  \| \| 1^e^ \| unknown \|  \| \| 1^d^* \| degarelix + enzalutamide \|  \| \| 1^f^* \| nivolumab + vemurafenib + ipilimumab \| AKI, P, hematuria  [9] \| \| 1^g^* \| denosumab + anastrozole \|  \| |
| --- | --- | --- | --- | --- | --- | --- | --- | --- | --- | --- | --- | --- | --- | --- | --- | --- | --- | --- | --- | --- | --- | --- | --- | --- | --- | --- | --- | --- | --- | --- | --- | --- | --- | --- | --- | --- | --- | --- | --- | --- | --- | --- | --- | --- | --- | --- | --- | --- | --- | --- | --- | --- | --- | --- | --- | --- | --- | --- | --- | --- | --- | --- | --- | --- | --- | --- | --- | --- | --- | --- | --- | --- | --- | --- | --- | --- | --- | --- | --- | --- | --- | --- | --- | --- | --- | --- | --- | --- | --- | --- | --- | --- | --- | --- | --- | --- | --- | --- | --- | --- | --- | --- | --- | --- | --- | --- | --- | --- | --- | --- | --- | --- | --- | --- | --- | --- | --- | --- |

Individual nephrotoxic potentials are described for the underlined molecule in the column situated before.

a, for stomach carcinoma; b, for small cell lung cancer; c, for cervical carcinoma; d, for prostate carcinoma (n=3); e, for pancreas carcinoma; f, for melanoma; g, for breast carcinoma. Radiotherapy was exclusively performed in kidney-sparing fields (cerebral, ossary or pelvic).

*, applied before diagnosis and therapy of the renal tumor.

AKI, acute kidney injury; CI, contra-indication; H, arterial hypertension; P, proteinuria; TI, tubulo-interstitial; TMA: thrombotic microangiopathy.

Table S2 – Linear regression analyses for eGFR at operation time – whole cohort

| **Prediction of eGFR at operation time** | | | | | | | | | | | |  |
| --- | --- | --- | --- | --- | --- | --- | --- | --- | --- | --- | --- | --- |
| *univariate linear regressions* | | | | | |  | *multivariate linear regression* | | | | |  |
|  | coef. | s.e | p-value | r2 |  |  |  | coef. | s.e | p-value |  |  |
| age (in years) | **-.69** | .103 | **<.001** | .186 |  |  | age (in years) | **-.649** | .095 | **<.001** |  |  |
| sex | -3.567 | 2.931 | .225 | .008 |  |  | arterial hypertension | -1.155 | 2.47 | .641 |  |  |
| arterial hypertension | **-6.085** | 2.97 | **.042** | .021 |  |  | vascular nephropathy | **-20.120** | 6.171 | **.001** |  |  |
| diabetes mellitus | -2.931 | 3.263 | .37 | .004 |  |  | glomerular scarring (in%) | **-.189** | .081 | **.021** |  |  |
| proteinuria | -1.916 | 4.528 | .673 | .001 |  |  | mesangial sclerosis grade=3 | -13.722 | 8.629 | .113 | vs. 0 |  |
| glomerulonephritis | 16.568 | 5.374 | .002 | .046 |  |  | interstitial fibrosis grade=3 | **-10.081** | 4.646 | **.031** | vs. 0 |  |
| interstitial nephritis | 4.769 | 5.67 | .401 | .004 |  |  | arteriosclerosis grade=3 | -4.482 | 2.671 | .095 | vs. 0 |  |
| vascular nephropathy | **-28.375** | 7.112 | **<.001** | .075 |  |  | **F(7,190)=16.171, p<.001, R2=.35** | | | | |  |
| glomerular scarring (in%) | **-.362** | .076 | **<.001** | .103 |  |  |  | | | | |  |
| mesangial sclerosis grade=1 | -3.256 | 3.175 | .306 | .005 | vs. 0 |  |  | |  |  |  |  |
| mesangial sclerosis grade=2 | **-16.865** | 8.408 | **.046** | .02 | vs. 0 |  |  | |  |  |  |  |
| mesangial sclerosis grade=3 | **-29.448** | 10.134 | **.004** | .041 | vs. 0 |  |  | |  |  |  |  |
| interstitial fibrosis grade=1 | -1.861 | 3.11 | .55 | .008 | vs. 0 |  |  | |  |  |  |  |
| interstitial fibrosis grade=2 | -6.299 | 5.663 | .267 | .006 | vs. 0 |  |  | |  |  |  |  |
| interstitial fibrosis grade=3 | **-18.241** | 4.546 | **<.001** | .076 | vs. 0 |  |  | |  |  |  |  |
| arteriosclerosis grade=1 | 9.429 | 3.18 | .003 | .043 | vs. 0 |  |  | |  |  |  |  |
| arteriosclerosis grade=2 | 1.263 | 2.993 | .674 | .001 | vs. 0 |  |  | |  |  |  |  |
| arteriosclerosis grade=3 | **-11.22** | 3.05 | **<.001** | .065 | vs. 0 |  |  | |  |  |  |  |
| arteriolosclerosis grade=1 | **-7.839** | 3.968 | **.05** | .015 | vs. 0 |  |  | |  |  |  |  |
| arteriolosclerosis grade=2 | -10.268 | 7.358 | .164 | .01 | vs. 0 |  |  | |  |  |  |  |
| arteriolosclerosis grade=3 | -15.324 | 7.808 | .051 | .019 | vs. 0 |  |  | |  |  |  |  |

coef., β-coefficient; s.e., standard error, r^2^ or R^2^, coefficient of determination.

Table S3 – Linear regression analyses for eGFR at operation time – malignant diseases

| **Prediction of eGFR at operation time** | | | | | | | | | | | |
| --- | --- | --- | --- | --- | --- | --- | --- | --- | --- | --- | --- |
| *univariate linear regressions* | | | | | |  | *multivariate linear regression* | | | | |
|  | coef. | s.e | p-value | r2 |  |  |  | coef. | s.e | p-value |  |
| age (in years) | **-.724** | .12 | **<.001** | .169 |  |  | age (in years) | **-.624** | .110 | **<.001** |  |
| sex | -3.284 | 3.044 | .282 | .006 |  |  | arterial hypertension | -1.97 | 2.603 | .450 |  |
| arterial hypertension | **-7.237** | 3.085 | **.02** | .03 |  |  | vascular nephropathy | **-14.762** | 6.597 | **.027** |  |
| diabetes mellitus | -3.102 | 3.323 | .352 | .005 |  |  | glomerular scarring (in%) | **-.201** | .087 | **.022** |  |
| proteinuria | .311 | 4.809 | .949 | 0 |  |  | arteriosclerosis grade=3 | -4.521 | 2.789 | .107 | vs. 0 |
| glomerulonephritis | 18.459 | 5.472 | <.001 | .058 |  |  | interstitial fibrosis grade=3 | **-10.977** | 5.385 | **.043** | vs. 0 |
| interstitial nephritis | -2.331 | 6.933 | .737 | .001 |  |  | arteriolosclerosis grade=1 | **-8.855** | 3.516 | **.013** | vs. 0 |
| vascular nephropathy | **-25.750** | 7.577 | **.001** | .061 |  |  | **F(7,173)=14.248, p<.001, R2=.366** | | | | |
| glomerular scarring (in%) | **-.395** | .078 | **<.001** | .127 |  |  |  |  |  |  |  |
| mesangial sclerosis grade=1 | -1.573 | 3.293 | .633 | .001 | vs. 0 |  |  |  |  |  |  |
| mesangial sclerosis grade=2 | -15.851 | 8.337 | .059 | .02 | vs. 0 |  |  |  |  |  |  |
| mesangial sclerosis grade=3 | **-28.455** | 10.031 | **.005** | .043 | vs. 0 |  |  |  |  |  |  |
| interstitial fibrosis grade=1 | -2.018 | 3.212 | .531 | .002 | vs. 0 |  |  |  |  |  |  |
| interstitial fibrosis grade=2 | -11.148 | 6.001 | .065 | .019 | vs. 0 |  |  |  |  |  |  |
| interstitial fibrosis grade=3 | **-23.082** | 4.871 | **<.001** | .111 | vs. 0 |  |  |  |  |  |  |
| arteriosclerosis grade=1 | 11.39 | 3.284 | <.001 | .063 | vs. 0 |  |  |  |  |  |  |
| arteriosclerosis grade=2 | -.946 | 3.112 | 761 | .001 | vs. 0 |  |  |  |  |  |  |
| arteriosclerosis grade=3 | **-10.476** | 3.134 | **.001** | .059 | vs. 0 |  |  |  |  |  |  |
| arteriolosclerosis grade=1 | **-12.441** | 4.128 | **.003** | .048 | vs. 0 |  |  |  |  |  |  |
| arteriolosclerosis grade=2 | -9.234 | 7.302 | .208 | .009 | vs. 0 |  |  |  |  |  |  |
| arteriolosclerosis grade=3 | -14.308 | 7.745 | .066 | .019 | vs. 0 |  |  |  |  |  |  |

coef., β-coefficient; s.e., standard error, r^2^ or R^2^, coefficient of determination.

Table S4 – Linear regression analyses for eGFR at operation time – clear cell renal cell carcinomas

| **Prediction of eGFR at operation time** | | | | | | | | | | | |
| --- | --- | --- | --- | --- | --- | --- | --- | --- | --- | --- | --- |
| *univariate linear regressions* | | | | | |  | *multivariate linear regressions* | | | | |
|  | coef. | s.e | p-value | r2 |  |  |  | coef. | s.e | p-value |  |
| age (in years) | **-.672** | .148 | **<.001** | .166 |  |  | age (in years) | **-.463** | .139 | **.001** |  |
| sex | -1.25 | 3.857 | .747 | .001 |  |  | arterial hypertension | -4.564 | 3.369 | .179 |  |
| arterial hypertension | **-10.689** | 3.770 | **.006** | .072 |  |  | interstitial nephritis | -7.284 | 7.184 | .313 |  |
| diabetes mellitus | -6.266 | 3.926 | .114 | .024 |  |  | glomerular scarring (in%) | -.145 | .144 | .316 |  |
| proteinuria | 1.993 | 6.194 | .749 | .002 |  |  | mesangial sclerosis grade=3 | -13.991 | 21.799 | .523 |  |
| glomerulonephritis | 26.55 | 8.578 | .003 | .085 |  |  | interstitial fibrosis grade=3 | **-18.367** | **8.428** | **.032** |  |
| interstitial nephritis | **-17.657** | 8.042 | **.03** | .045 |  |  | arteriosclerosis grade=3 | -5.244 | 3.581 | .146 |  |
| vascular nephropathy | -18.26 | 9.813 | .066 | .033 |  |  | arteriolosclerosis grade=3 | -8.537 | 9.628 | .377 |  |
| glomerular scarring (in%) | **-.475** | .118 | **<.001** | .135 |  |  | **F(8,96)=7.261, p<.001, R2=.377** | | | | |
| mesangial sclerosis grade=1 | -2.916 | 4.360 | .505 | .004 | vs. 0 |  |  |  |  |  |  |
| mesangial sclerosis grade=2 | **-23.314** | 11.231 | **.04** | .04 | vs. 0 |  |  |  |  |  |  |
| mesangial sclerosis grade=3 | **-46.76** | 19.116 | **.016** | .055 | vs. 0 |  |  |  |  |  |  |
| interstitial fibrosis grade=1 | -4.466 | 4.058 | .274 | .012 | vs. 0 |  |  |  |  |  |  |
| interstitial fibrosis grade=2 | -6.97 | 7.166 | .333 | .009 | vs. 0 |  |  |  |  |  |  |
| interstitial fibrosis grade=3 | **-36.571** | 7.397 | **<.001** | .192 | vs. 0 |  |  |  |  |  |  |
| arteriosclerosis grade=1 | 14.26 | 3.987 | <.001 | .11 | vs. 0 |  |  |  |  |  |  |
| arteriosclerosis grade=2 | -3.907 | 3.979 | .328 | .009 | vs. 0 |  |  |  |  |  |  |
| arteriosclerosis grade=3 | **-11.314** | 3.895 | **.004** | .076 | vs. 0 |  |  |  |  |  |  |
| arteriolosclerosis grade=1 | **-11.947** | 5.182 | **.023** | .049 | vs. 0 |  |  |  |  |  |  |
| arteriolosclerosis grade=2 | -6.843 | 11.444 | .551 | .003 | vs. 0 |  |  |  |  |  |  |
| arteriolosclerosis grade=3 | **-28.396** | 9.576 | **.004** | .079 | vs. 0 |  |  |  |  |  |  |

coef., β-coefficient; s.e., standard error, r^2^ or R^2^, coefficient of determination.

Table S5 – Linear regression analyses for eGFR at operation time – papillary tumors

| **Prediction of eGFR at operation time** | | | | | | | | | | | | |  |
| --- | --- | --- | --- | --- | --- | --- | --- | --- | --- | --- | --- | --- | --- |
| *univariate linear regressions* | | | | | |  | *multivariate linear regression* | | | | | |  |
|  | coef. | s.e | p-value | r2 |  |  |  | coef. | s.e | p-value | |  | |
| age (in years) | **-.69** | .31 | **.036** | .165 |  |  | age (in years) | **-.826** | .263 | **.005** | |  | |
| sex | 1.982 | 10.9 | .857 | .001 |  |  | vascular nephropathy | -21.49 | 23.24 | .365 | |  | |
| arterial hypertension | -1.771 | 8.766 | .842 | .002 |  |  | glomerular scarring (in%) | .19 | .1015 | .853 | |  | |
| diabetes mellitus | 14.042 | 13.185 | .297 | .043 |  |  | arteriosclerosis grade=3 | -46.495 | 87.631 | .598 | vs. 0 | | |
| proteinuria | -1.434 | 11.2 | .9 | .001 |  |  | **F(4,22)=5.175, p=.004, r2=.696** | | | | | | |
| glomerulonephritis | 12.536 | 10.615 | .249 | .053 |  |  |  | | | | | | |
| interstitial nephritis | 43.423 | 20.685 | .046 | .15 |  |  |  |  |  |  | |  | |
| vascular nephropathy | -41.731 | 20.824 | .056 | .138 |  |  |  |  |  |  | |  | |
| glomerular scarring (in%) | **-.366** | .131 | **.01** | .238 |  |  |  |  |  |  | |  | |
| mesangial sclerosis grade=1 | 9.151 | 9.096 | .324 | .039 | vs. 0 |  |  |  |  |  | |  | |
| mesangial sclerosis grade=2 | -28.28 | 15.156 | .074 | .122 | vs. 0 |  |  |  |  |  | |  | |
| mesangial sclerosis grade=3 | n.a | − | − | − | vs. 0 |  |  |  |  |  | |  | |
| interstitial fibrosis grade=1 | -5.853 | 8.695 | .507 | .018 | vs. 0 |  |  |  |  |  | |  | |
| interstitial fibrosis grade=2 | 33.038 | 21.439 | .136 | .087 | vs. 0 |  |  |  |  |  | |  | |
| interstitial fibrosis grade=3 | -14.167 | 9.789 | .16 | .077 | vs. 0 |  |  |  |  |  | |  | |
| arteriosclerosis grade=1 | -6.118 | 10.838 | .577 | .013 | vs. 0 |  |  |  |  |  | |  | |
| arteriosclerosis grade=2 | 19.194 | 7.889 | .022 | .191 | vs. 0 |  |  |  |  |  | |  | |
| arteriosclerosis grade=3 | **-32.833** | 11.774 | **.01** | .237 | vs. 0 |  |  |  |  |  | |  | |
| arteriolosclerosis grade=1 | -10.524 | 9.971 | .301 | .043 | vs. 0 |  |  |  |  |  | |  | |
| arteriolosclerosis grade=2 | -21.8 | 15.579 | .174 | .073 | vs. 0 |  |  |  |  |  | |  | |
| arteriolosclerosis grade=3 | n.a | − | − | − | vs. 0 |  |  |  |  |  | |  | |

coef., β-coefficient; s.e., standard error, r^2^ or R^2^, coefficient of determination, n.a., not available.

Table S6 – Linear regression analyses for eGFR at operation time – Neoplasms originating from intercalating cells of distal tubules

| **Prediction of eGFR at operation time** | | | | | | | | | | | |
| --- | --- | --- | --- | --- | --- | --- | --- | --- | --- | --- | --- |
| *univariate linear regressions* | | | | | |  | *multivariate linear regressions* | | | | |
|  | coef. | s.e | p-value | r2 |  |  |  | coef. | s.e | p-value |  |
| age (in years) | **-1.346** | .517 | **.019** | .297 |  |  | age (in years) | -.712 | .511 | .201 |  |
| sex | -13.092 | 11.662 | .278 | .073 |  |  | proteinuria* | **-50.223** | 16.142 | **.014** |  |
| arterial hypertension | -3.583 | 11.474 | .759 | .006 |  |  | **(a) F(2,8)=5.865, p=.027, R2=.595** | | | | |
| diabetes mellitus | 1.188 | 17.261 | .946 | 0 |  |  |  |  |  |  |  |
| proteinuria | **-50.5** | 16.964 | **.016** | .492 |  |  |  | coef. | s.e | p-value |  |
| glomerulonephritis | -3.118 | 23.672 | .897 | .001 |  |  | age (in years) | **-1.304** | .407 | **.006** |  |
| interstitial nephritis | n.a | − | − | − |  |  | glomerular scarring (in%) | **-1.047** | .318 | **.005** |  |
| vascular nephropathy | n.a | − | − | − |  |  | **(b) F(2,15)=10.9, p=.001, R2=.592** | | | | |
| glomerular scarring (in%) | **-1.079** | .399 | **.016** | .314 |  |  |  |  |  |  |  |
| mesangial sclerosis grade=1 | -10.167 | 11.225 | .379 | .049 | vs. 0 |  |  | coef. | s.e | p-value |  |
| mesangial sclerosis grade=2 | n.a | − | − | − | vs. 0 |  | age (in years) | **-1.326** | .397 | **.004** |  |
| mesangial sclerosis grade=3 | -14.765 | 23.396 | .537 | .024 | vs. 0 |  | interstitial fibrosis grade=3 | **-53.209** | 15.224 | **.003** | vs. 0 |
| interstitial fibrosis grade=1 | -7.954 | 11.948 | .515 | .027 | vs. 0 |  | **(c) F(2,15)=11.866, p=.001, R2=.613** | | | | |
| interstitial fibrosis grade=2 | n.a | − | − | − | vs. 0 |  |  | | | | |
| interstitial fibrosis grade=3 | **-53.941** | 19.471 | **.014** | .317 | vs. 0 |  |  |  |  |  |  |
| arteriosclerosis grade=1 | 8.083 | 11.33 | .486 | .031 | vs. 0 |  |  |  |  |  |  |
| arteriosclerosis grade=2 | 11.833 | 11.122 | .303 | .303 | vs. 0 |  |  |  |  |  |  |
| arteriosclerosis grade=3 | -19.917 | 10.376 | .073 | .187 | vs. 0 |  |  |  |  |  |  |
| arteriolosclerosis grade=1 | -7.25 | 17.168 | .678 | .011 | vs. 0 |  |  |  |  |  |  |
| arteriolosclerosis grade=2 | n.a | − | − | − | vs. 0 |  |  |  |  |  |  |
| arteriolosclerosis grade=3 | n.a | − | − | − | vs. 0 |  |  |  |  |  |  |

coef., β-coefficient; s.e., standard error, r^2^ or R^2^, coefficient of determination, n.a, not available. * proteinuria is collinear with glomerular scarring and interstitial fibrosis (variance inflation factor >10, respectively), hence models with proteinuria (a), glomerular scarring (b) or interstitial fibrosis grade=3 (c) have been computed.

Table S7 – Linear regression analyses for eGFR at operation time – benign diseases

| **Prediction of eGFR at operation time** | | | | | |
| --- | --- | --- | --- | --- | --- |
| *univariate linear regressions* | | | | | |
|  | coef. | s.e | p-value | r2 |  |
| age (in years) | -.407 | .5 | .437 | .069 |  |
| sex | -21.679 | 12.798 | .125 | .2442 |  |
| arterial hypertension | 9.433 | 13.847 | .513 | .049 |  |
| diabetes mellitus | 6.1 | 24.509 | .809 | .007 |  |
| proteinuria | -20.714 | 13.424 | .161 | .229 |  |
| glomerulonephritis | -1.6 | 24.588 | .95 | 0 |  |
| interstitial nephritis | 17.1 | 23.924 | .493 | .054 |  |
| vascular nephropathy | **-48.9** | 18.416 | **.026** | .439 |  |
| glomerular scarring (in%) | .18 | .421 | .679 | .02 |  |
| mesangial sclerosis grade=1 | -20.267 | 12.489 | .139 | .226 | vs. 0 |
| mesangial sclerosis grade=2 | n.a | − | − | − | vs. 0 |
| mesangial sclerosis grade=3 | n.a | − | − | − | vs. 0 |
| interstitial fibrosis grade=1 | -12.083 | 15.356 | .452 | .064 | vs. 0 |
| interstitial fibrosis grade=2 | 25.111 | 16.308 | .158 | .209 | vs. 0 |
| interstitial fibrosis grade=3 | -4.9 | 24.539 | .846 | .004 | vs. 0 |
| arteriosclerosis grade=1 | -25.857 | 11.905 | .058 | .344 | vs. 0 |
| arteriosclerosis grade=2 | 35.3 | 7.947 | .002 | .687 | vs. 0 |
| arteriosclerosis grade=3 | -33.5 | 21.912 | .161 | .206 | vs. 0 |
| arteriolosclerosis grade=1 | 21.375 | 14.186 | .166 | .201 | vs. 0 |
| arteriolosclerosis grade=2 | n.a |  |  |  | vs. 0 |
| arteriolosclerosis grade=3 | n.a |  |  |  | vs. 0 |

n.a., not available.

Table S8 – Linear regression analyses for eGFR slope – whole cohort

| **Prediction of ΔeGFR at 12 months** | | | | | |
| --- | --- | --- | --- | --- | --- |
| *univariate linear regressions* | | | | | |
|  | coef. | s.e | p-value | r2 |  |
| age (in years) | .041 | .46 | .928 | 0 |  |
| sex | -3.862 | 12.5 | .758 | .001 |  |
| arterial hypertension | 9.822 | 13.07 | .45 | .007 |  |
| diabetes mellitus | -1.9 | 14.621 | .894 | 0 |  |
| proteinuria | **-19.38** | 6.228 | **.003** | .15 |  |
| glomerulonephritis | -18.9 | 22.405 | .401 | .009 |  |
| interstitial nephritis | -5.951 | 22.493 | .792 | .001 |  |
| vascular nephropathy | 17.293 | 39.528 | .663 | .002 |  |
| glomerular scarring (in%) | .075 | .406 | .854 | 0 |  |
| mesangial sclerosis grade=1 | -11.615 | 13.397 | .313 | .013 | vs. 0 |
| mesangial sclerosis grade=2 | 9.378 | 57.309 | .87 | 0 | vs. 0 |
| mesangial sclerosis grade=3 | 18.716 | 40.726 | .647 | .003 | vs. 0 |
| interstitial fibrosis grade=1 | -4.625 | 13.963 | .741 | .001 | vs. 0 |
| interstitial fibrosis grade=2 | -20.129 | 33.429 | .549 | .004 | vs. 0 |
| interstitial fibrosis grade=3 | 6.3998 | 22.49 | .756 | .001 | vs. 0 |
| arteriosclerosis grade=1 | -8.53 | 14.854 | .567 | .004 | vs. 0 |
| arteriosclerosis grade=2 | -8.519 | 12.681 | .504 | .006 | vs. 0 |
| arteriosclerosis grade=3 | 18.069 | 13.196 | .175 | .023 | vs. 0 |
| arteriolosclerosis grade=1 | -6.087 | 18.431 | .742 | .001 | vs. 0 |
| arteriolosclerosis grade=2 | 15.597 | 32.454 | .976 | 0 | vs. 0 |
| arteriolosclerosis grade=3 | 1.296 | 40.779 | .975 | 0 | vs. 0 |

Table S9 – Linear regression analyses for eGFR slope for malignant tumors without potentially nephrotoxic oncologic therapy

| **Prediction of ΔeGFR at 12 months** | | | | | |
| --- | --- | --- | --- | --- | --- |
| *univariate linear regressions* | | | | | |
|  | coef. | s.e | p-value | r2 |  |
| age (in years) | .177 | .603 | .77 | .001 |  |
| sex | -7.56 | 13.098 | .57 | .004 |  |
| arterial hypertension | 13.25 | 13.884 | .343 | .012 |  |
| diabetes mellitus | -1.87 | 14.854 | .9 | 0 |  |
| proteinuria | **-14.457** | 7.098 | **.047** | .075 |  |
| glomerulonephritis | -19.265 | 24.675 | .437 | .008 |  |
| interstitial nephritis | -3.062 | 26.951 | .91 | 0 |  |
| vascular nephropathy | 15.422 | 41.741 | .713 | .002 |  |
| glomerular scarring (in%) | .095 | .316 | .765 | .001 |  |
| mesangial sclerosis grade=1 | -14.56 | 13.874 | .314 | .013 | vs. 0 |
| mesangial sclerosis grade=2 | 6.615 | 58.7 | .91 | 0 | vs. 0 |
| mesangial sclerosis grade=3 | 15.935 | 41.74 | .704 | .002 | vs. 0 |
| interstitial fibrosis grade=1 | -8.527 | 15.061 | .573 | .004 | vs. 0 |
| interstitial fibrosis grade=2 | 5.827 | 29.925 | .846 | 0 | vs. 0 |
| interstitial fibrosis grade=3 | 5.32 | 21.745 | .807 | .001 | vs. 0 |
| arteriosclerosis grade=1 | -11.688 | 16.68 | .487 | .006 | vs. 0 |
| arteriosclerosis grade=2 | -6.611 | 13.419 | .624 | .003 | vs. 0 |
| arteriosclerosis grade=3 | 16.55 | 13.39 | .22 | .019 | vs. 0 |
| arteriolosclerosis grade=1 | -68.093 | 19.715 | .683 | .002 | vs. 0 |
| arteriolosclerosis grade=2 | 13.719 | 34.299 | .69 | .002 | vs. 0 |
| arteriolosclerosis grade=3 | -1.506 | 41.777 | .971 | 0 | vs. 0 |

Figure legends

Figure S1 - Correlation plot of the patients’ age and the follow-up eGFR

Age (in years) correlated well with eGFR (ml/min/1.73m^2^) at 12 months follow-up (r=-.35, p<.001). The dotted line is the simple linear regression line (Y = -0,6732*X + 100,5).

Literature

1. Malyszko J, Kozlowska K, Kozlowski L, Malyszko J (2017) Nephrotoxicity of anticancer treatment. Nephrol Dial Transplant 32 (6):924-936. doi:10.1093/ndt/gfw338

2. American Society of Nephrology (2020) Chapter 11. Chemotherapy and Kidney Injury. <https://www.asn-online.org/education/distancelearning/curricula/onco/Chapter11.pdf>.

3. Lameire N (2014) Nephrotoxicity of recent anti-cancer agents. Clin Kidney J 7 (1):11-22. doi:10.1093/ckj/sft135

4. Takimoto T, Nakabori T, Osa A, Morita S, Terada H, Oseto S, Iwazawa T, Abe K (2012) Tubular nephrotoxicity induced by docetaxel in non-small-cell lung cancer patients. Int J Clin Oncol 17 (4):395-398. doi:10.1007/s10147-011-0304-5

5. Perazella MA (2012) Onco-nephrology: renal toxicities of chemotherapeutic agents. Clin J Am Soc Nephrol 7 (10):1713-1721. doi:10.2215/CJN.02780312

6. Sorich MJ, Rowland A, Kichenadasse G, Woodman RJ, Mangoni AA (2016) Risk factors of proteinuria in renal cell carcinoma patients treated with VEGF inhibitors: a secondary analysis of pooled clinical trial data. Br J Cancer 114 (12):1313-1317. doi:10.1038/bjc.2016.147

7. Motzer RJ, Nosov D, Eisen T, Bondarenko I, Lesovoy V, Lipatov O, Tomczak P, Lyulko O, Alyasova A, Harza M, Kogan M, Alekseev BY, Sternberg CN, Szczylik C, Cella D, Ivanescu C, Krivoshik A, Strahs A, Esteves B, Berkenblit A, Hutson TE (2013) Tivozanib versus sorafenib as initial targeted therapy for patients with metastatic renal cell carcinoma: results from a phase III trial. J Clin Oncol 31 (30):3791-3799. doi:10.1200/JCO.2012.47.4940

8. Wanchoo R, Karam S, Uppal NN, Barta VS, Deray G, Devoe C, Launay-Vacher V, Jhaveri KD, Cancer, Kidney International Network Workgroup on Immune Checkpoint I (2017) Adverse Renal Effects of Immune Checkpoint Inhibitors: A Narrative Review. Am J Nephrol 45 (2):160-169. doi:10.1159/000455014

9. Wanchoo R, Jhaveri KD, Deray G, Launay-Vacher V (2016) Renal effects of BRAF inhibitors: a systematic review by the Cancer and the Kidney International Network. Clin Kidney J 9 (2):245-251. doi:10.1093/ckj/sfv149
