# Supplementary figures and images for: More than ancillary records: clinical implications of renal pathology examination in tumor nephrectomy specimens
Source: J Nephrol. 2021 Apr 22;34(6):1833–44. doi: 10.1007/s40620-021-01030-0 (PMC8610937; doi:10.1007/s40620-021-01030-0)

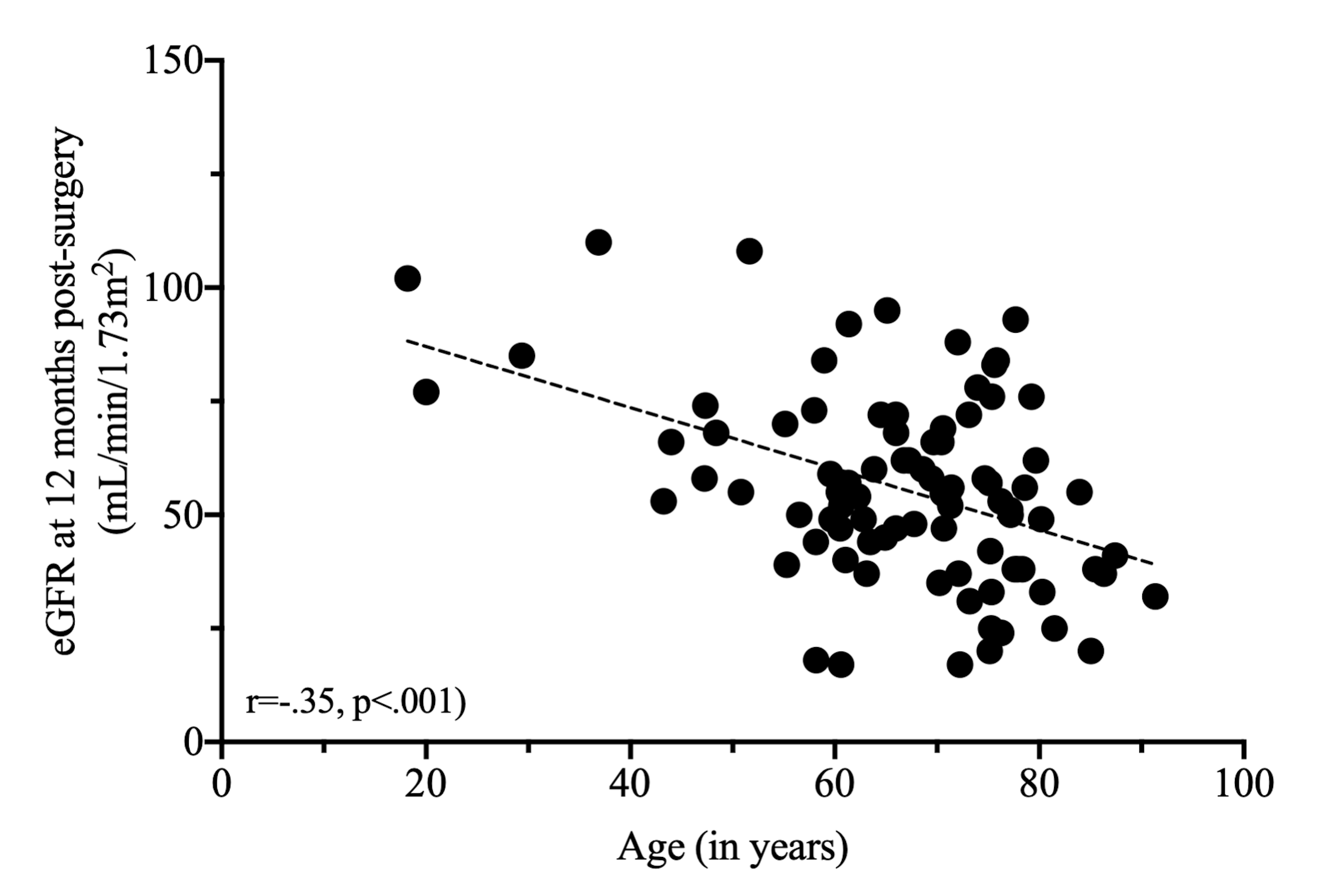

Supplement: Supplementary file 2 — Supplementary file2 (TIFF 23923 KB) [file 40620_2021_1030_MOESM2_ESM.tiff]
